# Postpartum Psychotropic Treatment Patterns and Breastfeeding: Nationwide Clustering Study

**DOI:** 10.1002/pds.70470

**Published:** 2026-08-30

**Authors:** Anders Hviid, Anna Laksafoss, Elisabeth O'Regan, Poul Videbech, Ingrid Bech Svalgaard

**Affiliations:** ^1^ Department of Data Science and AI in Health Statens Serum Institut Copenhagen Denmark; ^2^ PandemiX Center, Department of Science and Environment Roskilde University Roskilde Denmark; ^3^ Center for Neuropsychiatric Depression Research Mental Health Center Glostrup, Amager‐Hvidovre Hospital Denmark; ^4^ Department of Clinical Medicine University of Copenhagen Copenhagen Denmark

**Keywords:** breastfeeding, cluster analysis, pharmacoepidemiology, polypharmacy, postpartum period, psychotropic drugs, registers

## Abstract

**Introduction:**

Postpartum psychotropic medication use is heterogeneous, comprising continuation of prenatal treatment, initiation, transient use and complex polypharmacy. Prior studies have reported the prevalence of potential infant exposure to psychotropics via breast milk, but characterisations better capturing the heterogeneity of this exposure are lacking. We aimed to identify distinct postpartum psychotropic treatment patterns using hierarchical clustering accounting for medication type, timing and polypharmacy, and to describe breastfeeding characteristics across these patterns.

**Methods:**

Population‐based cohort study of mothers using psychotropics during the first postpartum year using linked Danish national registers 2012–2023. From a source population of 659 866 mother–child pairs with live‐born singleton births, 38 528 mothers redeemed at least one psychotropic prescription during the first postpartum year and comprised the study cohort. Weekly psychotropic use was defined by redeemed prescriptions using prescribed daily dose assumptions. Hierarchical agglomerative clustering (*tame* R package) identified distinct treatment patterns based on medication type, timing and polypharmacy. The number of patterns was determined from interpretability, size and examination of the hierarchical dendrogram. Exclusive breastfeeding prevalence and duration were described across patterns among pairs with recorded breastfeeding habits (64%).

**Results:**

Eight distinct treatment patterns were identified, differing in psychotropic type, timing and polypharmacy. Three SSRI‐dominated patterns (24 225 pairs, 63%) ranged from increasing SSRI use (Pattern I; *n* = 11 731), through continued SSRI use with high persistence (Pattern II; *n* = 7167), to mixed use involving SSRIs and other psychotropics (Pattern III; *n* = 5327; 48% using three or more medications). The remaining five patterns (14 303 pairs, 37%) were dominated by benzodiazepine‐related drugs, other antidepressants, centrally acting sympathomimetics, antipsychotics or complex mixed use, ranging from late‐initiated benzodiazepine‐related use (Pattern IV; median initiation Week 27; persistence 13%) to early‐initiated, highly persistent mixed use (Pattern VIII; median initiation Week 4; persistence 94%). Exclusive breastfeeding duration varied across patterns, with long‐duration exclusive breastfeeding (181 days or more) highest in the SSRI‐continued pattern (Pattern II, 10.6%) and lowest in the mixed‐use‐increasing and antipsychotics‐sporadic patterns (Patterns III and VII, 5.1% and 5.2%).

**Conclusion:**

Postpartum psychotropic treatment patterns are clinically heterogeneous and exclusive breastfeeding practices differ across treatment patterns.

## Key Points

1. Nationwide prescription data from 38 528 Danish mothers identified eight distinct patterns of psychotropic treatment during the first postpartum year.
2. Patterns differed in medication type, treatment timing, persistence and use of multiple medicines, revealing heterogeneity concealed by conventional exposed‐versus‐unexposed definitions.
3. Three SSRI‐dominated patterns comprised 63% of mothers; the remaining patterns involved benzodiazepine‐related drugs, other antidepressants, ADHD medicines, antipsychotics or complex mixed use.
4. Exclusive breastfeeding practices differed across patterns, with long‐duration breastfeeding least common in the mixed‐use‐increasing and antipsychotics‐sporadic patterns.
5. These differences are descriptive and cannot be attributed to medication use; treatment patterns may help identify groups needing individualised counselling and lactation support.

## Plain Language Summary

After childbirth, psychiatric treatment may continue from pregnancy, begin after delivery, last briefly or longer and involve one or several medicines. We used Danish national health records from 2012 to 2023 to study 38 528 mothers who filled at least one prescription for a psychiatric medicine during the first year after childbirth. We identified eight distinct treatment patterns based on the types of medicine used and when they were used. Three patterns, comprising 63% of the mothers, mainly involved selective serotonin reuptake inhibitors, a commonly used antidepressant. The other patterns mainly involved sedatives or medicines used to treat anxiety, depression, attention‐deficit/hyperactivity disorder, psychosis or more complex conditions requiring several medicines. Information on exclusive breastfeeding was available for 64% of the mothers. Breastfeeding duration differed across the eight patterns. These differences cannot show that psychiatric medicines caused mothers to breastfeed for shorter or longer periods because the groups also differed in psychiatric history and social characteristics, but they also do not allow such an effect to be ruled out. The findings may help identify mothers who could benefit from individualised treatment counselling and breastfeeding support.

## 1 Introduction

Psychiatric disorders affect a substantial proportion of women during the postpartum period, with prevalence estimates for depression ranging from 10% to 20% in the first year after delivery [1, 2]. Treatment with psychotropics is not uncommon, with estimates suggesting that up to 5% of women receive antidepressants in the postpartum period [3]. Because the recommended duration of exclusive breastfeeding is 6 months according to World Health Organization guidelines [4], mothers and clinicians face decisions about psychotropic use that involve potential infant exposure via breastfeeding [5]. The extent to which infants are exposed to psychotropics through breastfeeding depends on multiple factors, including the specific medication, dose, timing of administration relative to feeding, duration of both treatment and breastfeeding and milk composition [6]. Prior studies have quantified breast milk transfer for primarily antidepressants, finding low relative infant doses (< 10%) [6]. Epidemiological studies have reported on breastfeeding practices among women initiating or restarting antidepressants in the postpartum period, finding poorer breastfeeding outcomes compared with unexposed women [7].

Prior studies have largely treated psychotropic exposure as a binary classification (exposed versus unexposed during a defined period) or have examined single drug classes in isolation (mostly antidepressants). This simplification may obscure clinically relevant variation in how psychotropics are used, including differences in initiation timing (continuation from pregnancy versus postpartum initiation), persistence (transient versus sustained use), polypharmacy (monotherapy versus combinations) and medication types [8]. These patterns of use may have distinct implications for cumulative infant exposure through breastfeeding. Data‐driven approaches to exposure classification offer a method for identifying treatment patterns that reflect real‐world practice [9]. In this nationwide registry‐based cohort study of Danish mother–child pairs, we aimed to (1) identify distinct postpartum psychotropic treatment patterns using a hierarchical clustering approach that integrates medication type, timing, and polypharmacy and (2) describe exclusive breastfeeding characteristics across the identified patterns.

## 2 Materials and Methods

### 2.1 Study Design, Data Sources and Study Population

We conducted a nationwide registry‐based cohort study in Denmark linking individual‐level data across national registers using the unique personal ID assigned to all residents [10].

The Danish Medical Birth Register, comprising pregnancy and birth‐related information on all live‐ and stillborn children, was used to identify the source population of mother–child pairs [11]. Information on redeemed prescriptions was obtained from the Danish National Prescription Register, which captures all prescription drugs dispensed at Danish community pharmacies [12]. Hospital contacts and diagnoses were obtained from the Danish National Patient Register using International Classification of Diseases revision 10 (ICD‐10) codes [13]. Maternal socioeconomic and demographic characteristics, including place of birth, age, region of residence, family structure, level of education and equivalised household income at birth, were obtained from Statistics Denmark [14]. Exclusive breastfeeding duration was obtained from the Danish National Child Health Register, which collects data during routine visits from public health nurses offered to all mothers after delivery [15].

In Denmark, approval by an ethics committee is not required for register‐based research. The study was approved by Statens Serum Institut's Data Protection and Information Security Department.

We identified all live‐born singleton births in Denmark between January 1, 2012 and December 31, 2023. Mother–child pairs were excluded if the maternal birth date was missing or if the maternal region of residence at birth was not registered. Mothers redeeming at least one psychotropic prescription during the first postpartum year comprised the study cohort used for treatment pattern identification.

### 2.2 Psychotropic Medication Exposure

Psychotropic medications were defined using the following Anatomical Therapeutic Chemical (ATC) classification codes: antidepressants (N06A), hypnotics and sedatives (N05C), anxiolytics (N05B), antipsychotics (N05A), central nervous system stimulants (N06B) and antiepileptics (N03A). To restrict antiepileptic exposure to psychiatric indications, antiepileptic use was included only for mothers with a previous hospital contact for a manic episode or bipolar disorder (ICD‐10 codes F30 or F31).

For each mother–child pair, prescription data was included from 12 weeks before birth and throughout the first year after birth. The 12‐week period before birth was included to allow for prescriptions prior to birth to contribute to exposure in the earliest period after birth. Days covered for each redeemed prescription were estimated using the prescribed daily dose (PDD) (Table S2 in Supporting Information provides the specific values used in this study). For each filled prescription, the total number of days covered was calculated as: number of packages × number of units in package × (strength per unit/PDD). Thus, redeeming a prescription for a package of sertraline with 50 100 mg tablets corresponds to 50 days of sertraline exposure assuming a PDD of 100 mg. Exposure was further categorised as exposure during each of the 52 weekly intervals of the postpartum period.

### 2.3 Treatment Pattern Identification

In the study cohort, we identified treatment patterns using hierarchical agglomerative clustering implemented in the R package *tame* [9]. Hierarchical clustering assigns individuals to the same cluster if they are close together as defined by a bespoke distance measure. The *tame* approach uses a distance measure which both integrates relatedness based on the ATC hierarchy and relatedness of dose and timing profiles. In our context, where exposure is defined as yes/no on a weekly basis, we only consider timing profiles. The ATC system can be envisioned as a tree structure with the full ATC codes as the leaves. Two medications have shorter distances between them if they share common ancestors closer to the leaves of the tree structure than the root compared to other pairs of medications. The timing component compares weekly exposure status at each timepoint across the postpartum period and summarises differences over time using the Minkowski distance. The two components are combined multiplicatively, so that dissimilarities in exposure timing are amplified when medications are dissimilar by ATC codes. Clustering was performed using Ward's linkage method.

In hierarchical agglomerative clustering, the number of clusters cannot be determined by the data alone, but is defined by selecting a cut point in the dendrogram, and there is rarely a single “correct” choice. We therefore evaluated a set of candidate cluster solutions across different dendrogram cut heights and compared them with respect to (i) clinical interpretability of the resulting treatment patterns, (ii) separation of qualitatively distinct trajectories and (iii) cluster sizes to avoid unstable small groups. We selected an 8‐cluster solution because it represented the smallest number of clusters that still separated clinically distinct postpartum psychotropic treatment patterns, while maintaining adequate cluster sizes (minimum 750 mother–child pairs). The hierarchical agglomerative clustering was performed six times with different tuning of the medication ATC similarity and relative weighting of the medication exposure trajectory similarity. The tuning parameters are available in the Supporting Information (Table S1). The parameters selected were based on (i) clinical interpretability of the treatment patterns, (ii) separation of qualitatively distinct trajectories, (iii) separation of main groups of psychotropics.

### 2.4 Treatment Pattern Characteristics

For each identified treatment pattern, we presented the prevalences of specific psychotropics used and we characterised the exposure timing through median initiation timing, continuation status and persistence. Median initiation timing was defined as the median week of the first prescription in the postpartum period. Continuation status (yes/no) was defined by the prevalence of mothers redeeming a psychotropic prescription in the 12‐week period before birth. Persistence was defined as the prevalence of mothers with exposure in both the first and last 26 weeks of the postpartum period. Mixed use was characterised by the number of different psychotropics used and the median number of concurrent medications. Treatment patterns were further described according to the socioeconomic and demographic characteristics of the mothers.

Exclusive breastfeeding duration was categorised (30 days or less, 31–120 days, 121–180 days, more than 180 days or missing/no exclusive breastfeeding) for the subset of mothers with recorded breastfeeding practices in the Danish National Child Health Register [15]. For each treatment pattern, we characterised breastfeeding through the prevalence of any exclusive breastfeeding and the prevalence in the five different categories of breastfeeding duration above. Prevalence of breastfeeding characteristics were presented with 95% confidence intervals obtained by cluster‐stratified nonparametric bootstrap resampling (1000 samples).

Sensitivity analyses repeated clustering using 75% of the primary PDD values reflecting the fact that pregnant and breastfeeding women are plausibly sometimes prescribed slightly lower doses than those reflected by the PDD. Analyses were performed using R version 4.4.1 and the R package *tame* [9].

## 3 Results

### 3.1 Study Population

Of 696 845 live‐born singleton births recorded in the Danish Medical Birth Register between January 1, 2012 and December 31, 2023, we excluded 36 979 mother–child pairs due to not living in Denmark in the 2 years prior to birth or missing maternal birth date or unregistered maternal region of residence at birth, yielding 659 866 mother–child pairs. Among these, 38 528 (5.84%) mothers redeemed at least one psychotropic prescription during the first postpartum year and were included in the study cohort. Data on exclusive breastfeeding were available for 24 834 mother–child pairs with postpartum psychotropic use (64.5%).

### 3.2 Treatment Patterns

**FIGURE 1:**
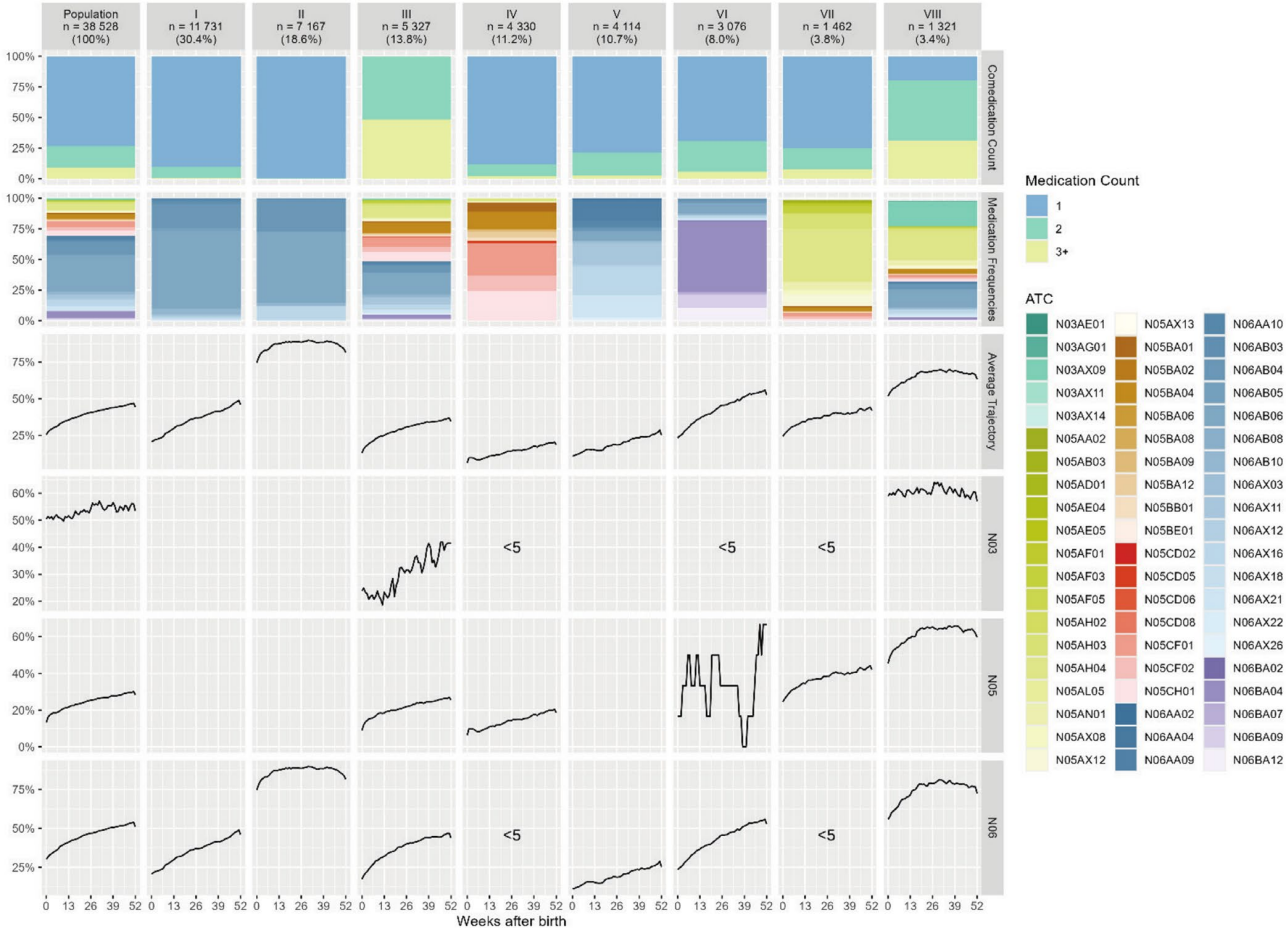
Characterisation of learned psychotropic treatment patterns in the first year post‐partum for the whole population (Column 1) and learned medication clusters (Column 2–9) by medication amount (Row 1), frequency of ATC codes used (Row 2), average exposure trajectory (Row 3) and average exposure trajectory by psychotropic group (Row 4–6). The average exposure trajectories are interpreted as the proportion of mother–child pairs exposed to a psychotropic at a given time. Trajectories and frequencies of ATC codes with less than five observations are not shown. Cluster I: SSRIs‐increasing, Cluster II: SSRIs‐continued, Cluster III: Mixed‐use‐increasing, Cluster IV: Benzodiazepine‐sporadic, Cluster V: Other‐antidepressants‐sporadic, Cluster VI: ADHD‐increasing, Cluster VII: Antipsychotics‐sporadic, Cluster VIII: Mixed‐use‐continued.

Hierarchical clustering identified eight distinct treatment patterns (Table 1, Figure 1). Each pattern was assigned a qualitative label summarising the drug class most commonly used, its average exposure trajectory across the first postpartum year and any mixed use. Three patterns, comprising 24 225 mother–child pairs (63%), were dominated by SSRI use. These ranged from increasing SSRI use with predominantly monotherapy (Pattern I, SSRIs‐increasing; *n* = 11 731; median initiation Week 16; persistence 49%), through continued SSRI use with high persistence (Pattern II, SSRIs‐continued; *n* = 7167; median initiation Week 6; continuation 77%; persistence 97%), to a mixed‐use pattern in which SSRIs were used together with other psychotropics (Pattern III, mixed‐use‐increasing; *n* = 5327; 48% using three or more medications).

**TABLE 1:** Medication use characteristics by treatment pattern (*N* = 38 528 mother/child‐pairs with postpartum psychotropic use, Denmark 2012–2023).

Treatment pattern	I	II	III	IV	V	VI	VII	VIII
	(*N* = 11 731)	(*N* = 7167)	(*N* = 5327)	(*N* = 4330)	(*N* = 4114)	(*N* = 3076)	(*N* = 1462)	(*N* = 1321)
Medication count								
1	90%	100%	0%	88%	79%	69%	75%	20%
2	9%	0%	52%	10%	19%	25%	18%	49%
3+	1%	0%	48%	2%	2%	6%	7%	31%
Dominant drug class								
Class	N06AB Selective Serotonin Reuptake Inhibitors	N06AB Selective Serotonin Reuptake Inhibitors	N06AB Selective Serotonin Reuptake Inhibitors	N05CF Benzodiaze‐pine related drugs	N06AX Other antidepress‐ants	N06BA Centrally acting sympatho‐mimetics	N05AH Diazepines, oxazepines, thiazepines and oxepines	N05AH Diazepines, oxazepines, thiazepines and oxepines
Prevalence	93%	87%	27%	39%	65%	82%	56%	27%
Timing and persistence								
Initiation week, median	16	6	10	27	20	12	14	4
Continuation	29%	77%	32%	4%	26%	32%	35%	80%
Persistence	49%	97%	70%	13%	41%	62%	46%	94%

*Note:* Cluster I: SSRIs‐increasing, Cluster II: SSRIs‐continued, Cluster III: Mixed‐use‐increasing, Cluster IV: Benzodiazepine‐sporadic, Cluster V: Other‐antidepressants‐sporadic, Cluster VI: ADHD‐increasing, Cluster VII: Antipsychotics‐sporadic, Cluster VIII: Mixed‐use‐continued.

The remaining five patterns (14 303 pairs; 37%) were each dominated by a different non‐SSRI psychotropic. Three reflected lower‐persistence or more intermittent use: benzodiazepine‐related anxiolytics and hypnotics initiated late and rarely persisting (Pattern IV, benzodiazepine‐sporadic; *n* = 4330; median initiation Week 27; persistence 13%), non‐SSRI antidepressants with moderate persistence (Pattern V, other‐antidepressants‐sporadic; *n* = 4114; persistence 41%) and antipsychotic‐dominated use with moderate persistence (Pattern VII, antipsychotics‐sporadic; *n* = 1462; persistence 46%). Two reflected sustained or increasing treatment: centrally acting sympathomimetics with increasing use throughout the postpartum period (Pattern VI, ADHD‐increasing; *n* = 3076) and a complex, highly persistent mixed‐use pattern (Pattern VIII, mixed‐use‐continued; *n* = 1321; median initiation Week 4; persistence 94%).

Weekly average exposure trajectories for any psychotropic, antiepileptics (ATC N03), psycholeptics (N05) and psychoanaleptics (N06) are presented in Figure 1. Pattern II (SSRIs‐continued) and Pattern VIII (mixed‐use‐continued) showed high coverage from birth, consistent with continuation of prenatal treatment, with persistent treatment throughout the postpartum period. Patterns I (SSRIs‐increasing), III (mixed‐use‐increasing), V (other‐antidepressants‐sporadic), VI (ADHD‐increasing) and VII (antipsychotics‐sporadic) showed rising coverage throughout the postpartum year. Pattern IV (benzodiazepine‐sporadic) showed lower coverage consistent with transient use across many different individuals.

**FIGURE 2:**
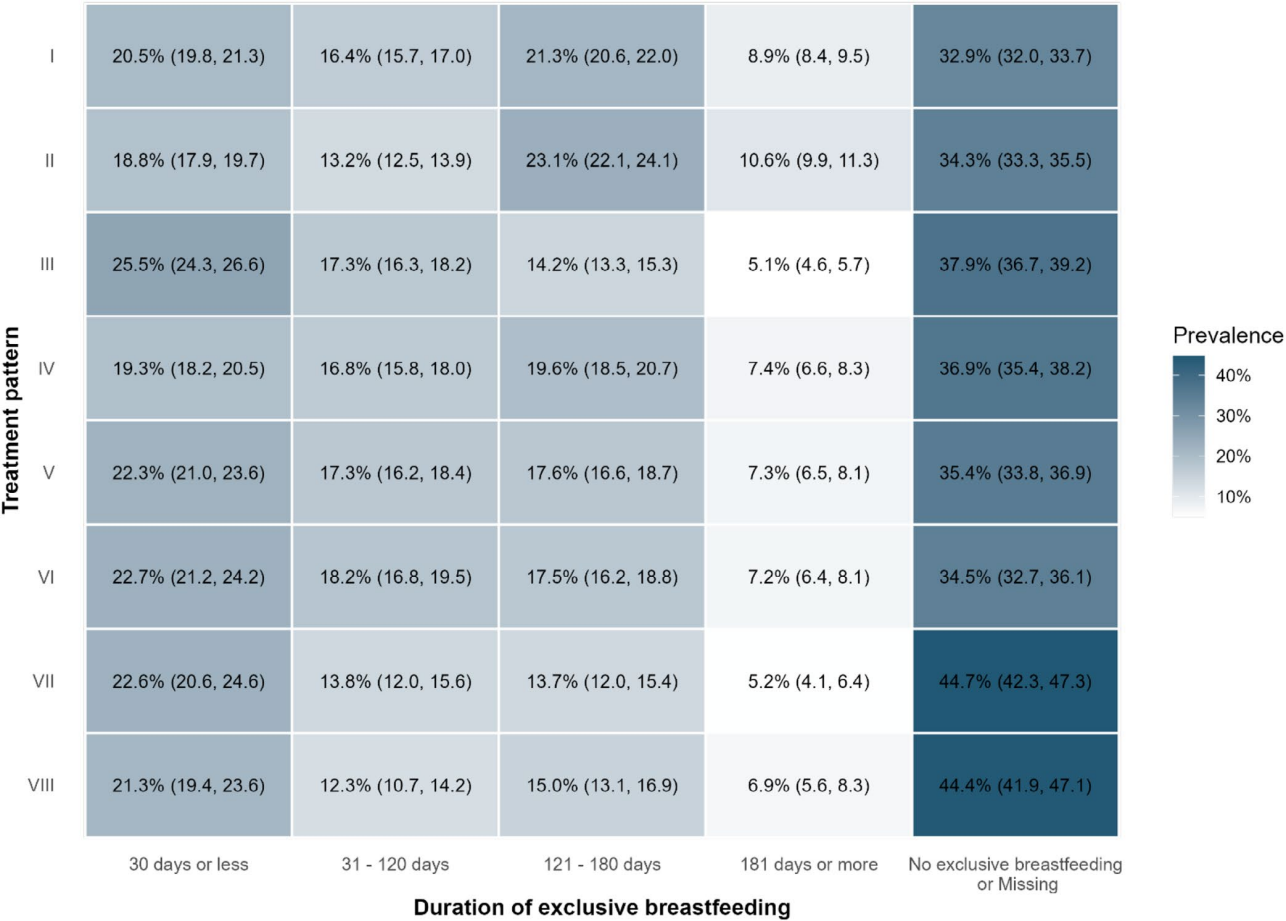
Prevalence and 95% confidence intervals of exclusive breastfeeding duration categories by treatment pattern. Cluster I: SSRIs‐increasing, Cluster II: SSRIs‐continued, Cluster III: Mixed‐use‐increasing, Cluster IV: Benzodiazepine‐sporadic, Cluster V: Other‐antidepressants‐sporadic, Cluster VI: ADHD‐increasing, Cluster VII: Antipsychotics‐sporadic, Cluster VIII: Mixed‐use‐continued.

Clustering with 0.75 PDD assumptions yielded similar psychotropic treatment patterns (Figure 2).

### 3.3 Maternal Characteristics of the Treatment Patterns

Maternal characteristics differed markedly across patterns (Table 2). A psychiatric hospital diagnosis in the five years before birth ranged from least common in the benzodiazepine‐sporadic pattern (IV, 20.0%) to most common in the mixed‐use‐continued (VIII, 85.6%) and antipsychotics‐sporadic (VII, 68.6%) patterns. Diagnoses mirrored the dominant drug classes: manic episode and bipolar disorder were most common in the mixed‐use‐continued pattern (VIII, 46.7%), schizophrenia and other psychotic disorders were concentrated in the antipsychotics‐sporadic pattern (VII, 24.8%), childhood‐onset behavioural and emotional disorders characterised the ADHD‐increasing pattern (VI, 37.4%), and depression was frequent in the mixed‐use‐continued (VIII, 33.8%) and SSRIs‐continued (II, 29.5%) patterns.

**TABLE 2:** Demographic characteristics by treatment pattern (*N* = 38 528 mother/child‐pairs with postpartum psychotropic use, Denmark 2012–2023).

	I	II	III	IV	V	VI	VII	VIII
	(*N* = 11 731)	(*N* = 7167)	(*N* = 5327)	(*N* = 4330)	(*N* = 4114)	(*N* = 3076)	(*N* = 1462)	(*N* = 1321)
Maternal age at birth								
Younger than 25	1357 (11.6%)	527 (7.4%)	640 (12.0%)	398 (9.2%)	457 (11.1%)	644 (20.9%)	245 (16.8%)	90 (6.8%)
25–29	3545 (30.2%)	1992 (27.8%)	1526 (28.6%)	1121 (25.9%)	1260 (30.6%)	1131 (36.8%)	445 (30.4%)	390 (29.5%)
30–34	4093 (34.9%)	2777 (38.7%)	1801 (33.8%)	1572 (36.3%)	1393 (33.9%)	868 (28.2%)	447 (30.6%)	472 (35.7%)
35–39	2175 (18.5%)	1507 (21.0%)	1054 (19.8%)	960 (22.2%)	790 (19.2%)	355 (11.5%)	246 (16.8%)	290 (22.0%)
40 or older	561 (4.8%)	364 (5.1%)	306 (5.7%)	279 (6.4%)	214 (5.2%)	78 (2.5%)	79 (5.4%)	79 (6.0%)
Maternal region of residence								
Capital Region	3389 (28.9%)	2143 (29.9%)	1522 (28.6%)	1710 (39.5%)	863 (21.0%)	663 (21.6%)	389 (26.6%)	433 (32.8%)
Region Zealand	1373 (11.7%)	723 (10.1%)	654 (12.3%)	551 (12.7%)	518 (12.6%)	402 (13.1%)	200 (13.7%)	116 (8.8%)
Region of Southern Denmark	2482 (21.2%)	1534 (21.4%)	1063 (20.0%)	710 (16.4%)	1037 (25.2%)	555 (18.0%)	445 (30.4%)	275 (20.8%)
Region of Central Denmark	3262 (27.8%)	2072 (28.9%)	1545 (29.0%)	1014 (23.4%)	1205 (29.3%)	1044 (33.9%)	304 (20.8%)	379 (28.7%)
Region of Northern Denmark	1215 (10.4%)	≤ 695 (9.7%)	≤ 543 (10.2%)	≤ 345 (7.9%)	≤ 491 (11.9%)	≤ 412 (13.4%)	≤ 124 (8.5%)	≤ 118 (9.0%)
Cannot be categorised	10 (0.1%)	≤ 5 (0.0%)	≤ 5 (0.0%)	≤ 5 (0.0%)	≤ 5 (0.0%)	≤ 5 (0.0%)	≤ 5 (0.0%)	≤ 5 (0.0%)
Maternal place of birth								
Denmark	10 176 (86.7%)	6611 (92.2%)	4586 (86.1%)	3572 (82.5%)	3468 (84.3%)	2912 (94.7%)	1214 (83.0%)	1204 (91.1%)
Europe	835 (7.1%)	322 (4.5%)	356 (6.7%)	366 (8.5%)	286 (7.0%)	83 (2.7%)	110 (7.5%)	59 (4.5%)
Outside Europe	720 (6.1%)	234 (3.3%)	385 (7.2%)	392 (9.1%)	360 (8.8%)	81 (2.6%)	138 (9.4%)	58 (4.4%)
Disposable household income								
Low	3691 (31.5%)	1982 (27.7%)	1850 (34.7%)	1106 (25.5%)	1505 (36.6%)	1304 (42.4%)	703 (48.1%)	473 (35.8%)
Middle	4097 (34.9%)	2589 (36.1%)	1694 (31.8%)	1252 (28.9%)	1397 (34.0%)	1052 (34.2%)	413 (28.2%)	454 (34.4%)
High	3943 (33.6%)	2596 (36.2%)	1783 (33.5%)	1972 (45.5%)	1212 (29.5%)	720 (23.4%)	346 (23.7%)	394 (29.8%)
Maternal education								
Primary	12 (0.1%)	≤ 5 (0.0%)	14 (0.3%)	12 (0.3%)	14 (0.3%)	≤ 5 (0.0%)	≤ 5 (0.0%)	0 (0%)
Lower secondary	2291 (19.5%)	1138 (15.9%)	1273 (23.9%)	720 (16.6%)	1008 (24.5%)	1131 (36.8%)	559 (38.2%)	314 (23.8%)
Upper secondary	4040 (34.4%)	2364 (33.0%)	1699 (31.9%)	1234 (28.5%)	1528 (37.1%)	1036 (33.7%)	414 (28.3%)	423 (32.0%)
Short cycle tertiary	548 (4.7%)	319 (4.5%)	179 (3.4%)	215 (5.0%)	159 (3.9%)	92 (3.0%)	47 (3.2%)	47 (3.6%)
Bachelor or equivalent	2955 (25.2%)	2026 (28.3%)	1310 (24.6%)	1111 (25.7%)	838 (20.4%)	544 (17.7%)	251 (17.2%)	328 (24.8%)
Master or equivalent	1665 (14.2%)	1225 (17.1%)	725 (13.6%)	944 (21.8%)	468 (11.4%)	234 (7.6%)	153 (10.5%)	188 (14.2%)
Doctoral or equivalent	85 (0.7%)	62 (0.9%)	61 (1.1%)	45 (1.0%)	15 (0.4%)	7 (0.2%)	8 (0.5%)	≤ 10 (0.7%)
Not elsewhere classified	22 (0.2%)	≤ 10 (0.1%)	14 (0.3%)	16 (0.4%)	24 (0.6%)	≤ 5 (0.0%)	9 (0.6%)	≤ 5 (0.0%)
Missing	113 (1.0%)	23 (0.3%)	52 (1.0%)	33 (0.8%)	60 (1.5%)	27 (0.9%)	21 (1.4%)	11 (0.8%)
Parity								
1	5106 (43.5%)	3243 (45.2%)	2488 (46.7%)	2038 (47.1%)	1698 (41.3%)	1528 (49.7%)	777 (53.1%)	721 (54.6%)
2	4487 (38.2%)	2769 (38.6%)	1846 (34.7%)	1551 (35.8%)	1451 (35.3%)	1062 (34.5%)	432 (29.5%)	414 (31.3%)
3	1525 (13.0%)	852 (11.9%)	666 (12.5%)	501 (11.6%)	640 (15.6%)	330 (10.7%)	151 (10.3%)	127 (9.6%)
4 or more	526 (4.5%)	283 (3.9%)	294 (5.5%)	212 (4.9%)	296 (7.2%)	139 (4.5%)	95 (6.5%)	≤ 59 (4.5%)
Unknown	87 (0.7%)	20 (0.3%)	33 (0.6%)	28 (0.6%)	29 (0.7%)	17 (0.6%)	7 (0.5%)	≤ 5 (0.0%)
Psychiatric hospital diagnosis in the 5 years before birth								
No	7494 (63.9%)	3404 (47.5%)	2711 (50.9%)	3462 (80.0%)	2706 (65.8%)	1482 (48.2%)	459 (31.4%)	190 (14.4%)
Yes	4237 (36.1%)	3763 (52.5%)	2616 (49.1%)	868 (20.0%)	1408 (34.2%)	1594 (51.8%)	1003 (68.6%)	1131 (85.6%)
Type of psychiatric hospital diagnosis in the 5 years before birth								
Disorders of adult personality and behaviour	777 (6.6%)	662 (9.2%)	638 (12.0%)	198 (4.6%)	291 (7.1%)	334 (10.9%)	253 (17.3%)	264 (20.0%)
Anxiety, dissociative, stress‐related, somatoform and other nonpsychotic mental disorder	2445 (20.8%)	2121 (29.6%)	1452 (27.3%)	501 (11.6%)	743 (18.1%)	594 (19.3%)	457 (31.3%)	412 (31.2%)
Mental disorder, not otherwise specified	344 (2.9%)	290 (4.0%)	239 (4.5%)	73 (1.7%)	122 (3.0%)	144 (4.7%)	137 (9.4%)	114 (8.6%)
Depression	1976 (16.8%)	2113 (29.5%)	1204 (22.6%)	211 (4.9%)	749 (18.2%)	387 (12.6%)	218 (14.9%)	447 (33.8%)
Schizophrenia, schizotypal, delusional and other non‐mood psychotic disorders	143 (1.2%)	93 (1.3%)	283 (5.3%)	87 (2.0%)	48 (1.2%)	53 (1.7%)	362 (24.8%)	146 (11.1%)
Self‐harming behaviours	76 (0.6%)	53 (0.7%)	106 (2.0%)	22 (0.5%)	37 (0.9%)	40 (1.3%)	43 (2.9%)	23 (1.7%)
Manic episode and bipolar episode	72 (0.6%)	56 (0.8%)	211 (4.0%)	72 (1.7%)	32 (0.8%)	29 (0.9%)	202 (13.8%)	617 (46.7%)
Eating disorders	358 (3.1%)	319 (4.5%)	239 (4.5%)	97 (2.2%)	107 (2.6%)	110 (3.6%)	63 (4.3%)	82 (6.2%)
Behavioural and emotional disorders with onset usually occurring during childhood and adolescence	219 (1.9%)	166 (2.3%)	405 (7.6%)	68 (1.6%)	88 (2.1%)	1151 (37.4%)	122 (8.3%)	109 (8.3%)
Mental and behavioural disorder due to psychoactive substance use	423 (3.6%)	272 (3.8%)	352 (6.6%)	163 (3.8%)	195 (4.7%)	233 (7.6%)	180 (12.3%)	127 (9.6%)
Pervasive and specific developmental disorders	75 (0.6%)	54 (0.8%)	96 (1.8%)	19 (0.4%)	26 (0.6%)	207 (6.7%)	37 (2.5%)	25 (1.9%)
Intellectual disabilities	32 (0.3%)	19 (0.3%)	46 (0.9%)	13 (0.3%)	16 (0.4%)	32 (1.0%)	28 (1.9%)	20 (1.5%)
Persistent mood disorders and unspecified mood disorder	87 (0.7%)	103 (1.4%)	71 (1.3%)	10 (0.2%)	28 (0.7%)	17 (0.6%)	16 (1.1%)	41 (3.1%)

*Note:* Cluster I: SSRIs‐increasing, Cluster II: SSRIs‐continued, Cluster III: Mixed‐use‐increasing, Cluster IV: Benzodiazepine‐sporadic, Cluster V: Other‐antidepressants‐sporadic, Cluster VI: ADHD‐increasing, Cluster VII: Antipsychotics‐sporadic, Cluster VIII: Mixed‐use‐continued.

The ADHD‐increasing (VI) and antipsychotics‐sporadic (VII) patterns comprised the youngest mothers (21% and 17% under 25) with the lowest educational attainment (lower‐secondary education 37% and 38%) and highest proportions in the lowest income tertile (42% and 48%). In contrast, the benzodiazepine‐sporadic pattern (IV) had the highest proportions of mothers with tertiary education and high household income and was most concentrated in the Capital Region (40%). First‐time mothers predominated in the antipsychotics‐sporadic (VII, 53%) and mixed‐use‐continued (VIII, 55%) patterns.

### 3.4 Breastfeeding Across Treatment Patterns

**FIGURE 3:**
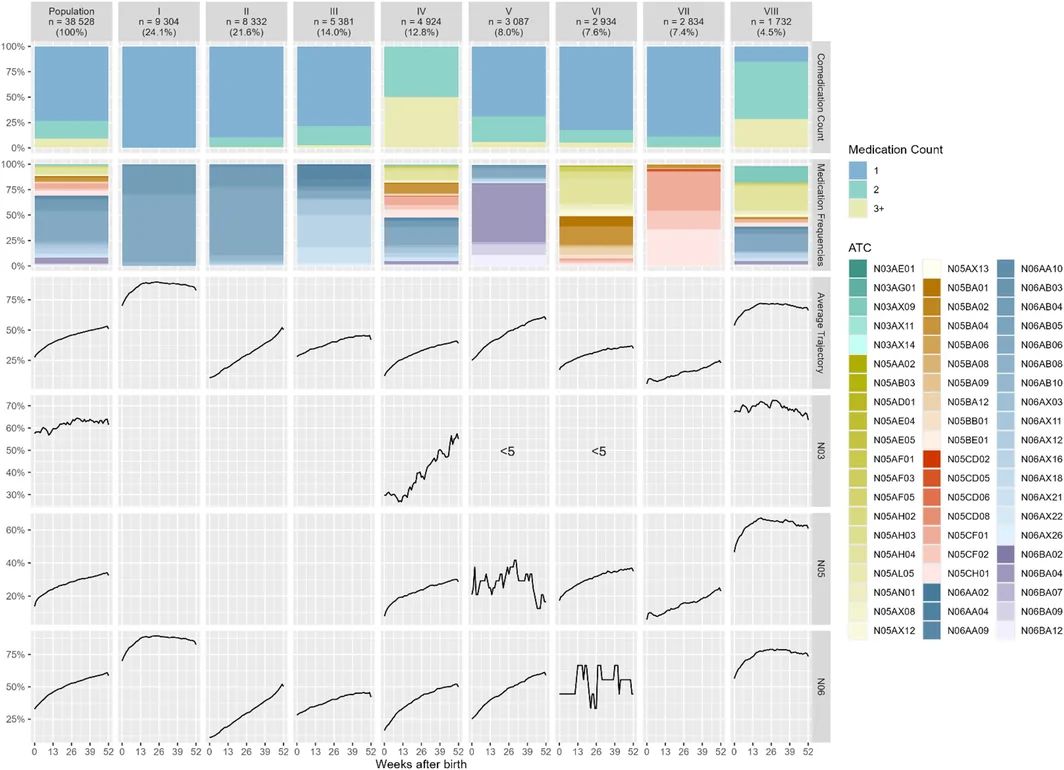
Characterisation of learned psychotropic treatment patterns with prescribed daily dose at 75% in the first year post‐partum for the whole population (Column 1) and learned medication clusters (Column 2–9) by medication amount (Row 1), frequency of ATC codes used (Row 2), average exposure trajectory (Row 3) and average exposure trajectory by psychotropic group (Row 4–6). The average exposure trajectories are interpreted as the proportion of mother–child pairs exposed to a psychotropic at a given time. Trajectories and frequencies of ATC codes with less than five observations are not shown. Cluster I: SSRIs‐continued, Cluster II: SSRIs‐increasing, Cluster III: Other‐antidepressants‐continued, Cluster IV: Mixed‐use‐increasing, Cluster V: ADHD‐increasing, Cluster VI: Antipsychotics‐and‐anxiolytics‐sporadic, Cluster VII: Benzodiazepine‐sporadic, Cluster VIII: Mixed‐use‐continued.

Among mothers with postpartum psychotropic use, the distribution of exclusive breastfeeding duration varied across treatment patterns (Figure 3). Short exclusive breastfeeding (30 days or less) was most common in the mixed‐use‐increasing pattern (III, 25.5%) and least common in the SSRI‐continued pattern (II, 18.8%), while sustained exclusive breastfeeding (181 days or more) was highest in the SSRI‐continued pattern (II, 10.6%) and lowest in the mixed‐use‐increasing (III, 5.1%) and antipsychotics‐sporadic (VII, 5.2%) patterns. The proportion with no exclusive breastfeeding or missing data was lowest in the SSRI‐increasing pattern (I, 32.9%) and highest in the antipsychotics‐sporadic (VII, 44.7%) and mixed‐use‐continued (VIII, 44.4%) patterns.

## 4 Discussion

In this nationwide cohort of 38 528 Danish mother–child pairs with postpartum psychotropic use, hierarchical clustering of medication type, timing and polypharmacy identified eight treatment patterns. Three SSRI‐dominated patterns comprised 24 225 pairs (63%), ranging from increasing SSRI use, through continued SSRI use with high persistence, to a mixed‐use pattern in which SSRIs were present alongside frequent mixed use. The remaining five patterns (14 303 pairs, 37%) were defined by benzodiazepine‐related drugs, other antidepressants, centrally acting sympathomimetics, antipsychotic‐dominated use or complex mixed use. They differed in initiation timing, persistence and mixed use, from late‐initiated benzodiazepine‐related use to early‐initiated, highly persistent mixed use. Exclusive breastfeeding differed across treatment patterns: long‐duration breastfeeding (181 days or more) was most common in the SSRI‐continued pattern (10.6%) and least common in the mixed‐use‐increasing and antipsychotics‐sporadic patterns (5.1% and 5.2%), and the proportion with no or unrecorded exclusive breastfeeding was highest in the antipsychotics‐sporadic and mixed‐use‐continued patterns.

Most previous studies of postpartum psychotropic treatment have used binary (ever vs. never) single‐class definitions over a fixed time window. These definitions discard information on timing, duration, persistence, switching and co‐medication, and can misclassify exposure [8]. Longitudinal methods that model individual medication trajectories, such as group‐based trajectory models and longitudinal k‐means clustering, have been applied to perinatal antidepressant use and capture these features better [16, 17]. The *tame* approach extends this work by combining medication type, timing and concurrent use in a single ATC‐informed distance measure. This is its first application to postpartum psychotropic use, and it shows heterogeneity in initiation, persistence, and polypharmacy that binary classifications cannot capture.

An earlier study from Norway has reported initiated or restarted postpartum use of antidepressants were associated with lower rates of any and exclusive breastfeeding and abrupt discontinuation compared to a control group with self‐reported mental health issues but no antidepressant use, whereas use continued from pregnancy differed little [7]. A recent meta‐analysis of women using medicines in pregnancy or postpartum found high breastfeeding initiation but lower continuation at 6 months, with antidepressants and antiepileptics among the drugs associated with markedly reduced continuation [18]. In Denmark, about 2% of exclusively breastfed infants are potentially exposed to psychotropics through breast milk, mainly antidepressants and particularly sertraline [19]. Our findings suggests that breastfeeding practices differed not only between SSRI‐ and non‐SSRI‐dominated patterns, but also among the SSRI‐dominated patterns themselves.

Several mechanisms may explain the differences in breastfeeding practices across treatment patterns. Patterns marked by continuation from pregnancy and use throughout the postpartum period, such as the SSRIs‐continued and mixed‐use‐continued patterns, may reflect more severe or persistent illness, which could affect breastfeeding through symptom burden or clinical advice. Patterns with increasing or later postpartum use, such as the SSRIs‐increasing, ADHD‐increasing and benzodiazepine‐sporadic patterns, may reflect conditions arising or resuming after breastfeeding was established. However, the descriptive, cross‐sectional design of our study does not allow for causal inference.

The treatment patterns differed by socio‐demographic characteristics. The ADHD‐increasing and antipsychotics‐sporadic patterns had the youngest mothers, the lowest educational attainment and the highest proportions in the lowest income tertile, whereas the benzodiazepine‐sporadic pattern had the highest proportions with tertiary education and high income. Because maternal age, education and income are themselves associated with breastfeeding, differences in breastfeeding across treatment patterns cannot be attributed to medication use itself.

The variation in breastfeeding across treatment patterns suggests that subgroups exist, such as the mixed‐use‐increasing and antipsychotics‐sporadic patterns with the lowest prevalence of long‐duration exclusive breastfeeding and the antipsychotics‐sporadic and mixed‐use‐continued patterns with the highest prevalence of no or missing exclusive breastfeeding that may benefit from additional lactation support, given the benefits of longer breastfeeding for child development [20]. The findings do not indicate that any pattern contraindicates breastfeeding. Relative infant doses are low for most psychotropics [21] but not all [22] and decisions should remain individual and depend on the medication, the underlying condition and maternal circumstances.

The study has several strengths. The nationwide linked design resulted in complete ascertainment of redeemed prescriptions and limited selection bias. The clustering approach combined medication type, timing and polypharmacy in one classification, avoiding the information loss of binary definitions and produced clinically interpretable patterns. Register‐based prescription and breastfeeding data also avoided recall bias.

A number of limitations exist. Redeemed prescriptions indicate dispensing, not ingestion and dose estimates based on prescribed daily dose assumptions may not reflect prescribed or actual consumption [23]. Reassuringly, a sensitivity analysis using 0.75 prescribed daily doses gave similar patterns. It is plausible that some mothers will be extra cautious during breastfeeding and not fully use a dispensed prescription. This is more likely to be mothers with less severe conditions and fewer symptoms and should be taken into account when interpreting the results. The restriction for antiepileptic use to be combined with a previous hospital contact is restrictive and may miss mothers diagnosed exclusively in primary care. Breastfeeding data covered only a subset of mothers and recorded exclusive breastfeeding in categorical intervals, without information on partial breastfeeding. Since not all mothers in the study had breastfeeding recordings in the Danish National Child Health Register and absence of a breastfeeding recording may reflect either lack of registration or no full breastfeeding, selection and/or misclassification of breastfeeding status cannot be excluded. We used a cross‐sectional design, which does not assess the temporal overlap between medication use and breastfeeding. The patterns are data‐driven and a different number of clusters or parameters could give different solutions. Finally, there was no non‐user comparison group.

## 5 Conclusion

Postpartum psychotropic treatment in this cohort was heterogeneous in medication type, timing and polypharmacy, and exclusive breastfeeding practices differed across treatment patterns. Our findings support the understanding of real‐world psychotropic use in the postpartum period. Future studies could link these patterns to infant outcomes using methods that take the longitudinal overlap between medication use and breastfeeding into account.

## Author Contributions

A.H. and E.O. conceived and designed the study. A.H. drafted the manuscript. I.B.S. performed the statistical analyses. All authors interpreted the data and critically revised the manuscript and approved the final version.

## Funding

Independent Research Fund Denmark 3166‐00022B. The funder had no role in the design of the study, the analysis or interpretation of data or the writing of the manuscript.

## Ethics Statement

In Denmark, register‐based research does not require approval from an ethics committee, and informed consent is not required for studies based solely on pseudonymised register data. The study was approved by Statens Serum Institut's Data Protection and Information Security Department.

## Conflicts of Interest

A.H. reports funding from Novo Nordisk Foundation, Lundbeck Foundation and Sundhedsdonationer outside the submitted work.

## Supporting information


**Table S1:** Tuning parameters for the six hierarchical cluster analyses (HCA) performed. The theta parameters, $\theta =\left(\theta_{0},\theta_{1},\theta_{2},\theta_{3},\theta_{4},\theta_{5}\right)$ encodes the relative importance of each ATC level. This study only includes psychotropics therefore only the tuning of $\theta_{2},\theta_{3},\theta_{4},\theta_{5}$ is important. It is also possible to tune the HCA by gamma (*γ*), which controls the importance of the dose‐timing aspect and the ATC of the psychotropic treatment pattern. A larger *γ* gives more weight to the timing aspect.
**Table S2:** Prescribed daily doses used in the study.

## Data Availability

The individual‐level data for this study is sourced from Danish national health and administrative registers and cannot be shared publicly due to Danish data protection legislation. Access to the registers can be obtained by application to the Danish Health Data Authority and Statistics Denmark, subject to the required approvals. The *tame* R package is freely available on CRAN. Analysis code is available from the authors on reasonable request.
