# Supplementary material for: Postpartum Psychotropic Treatment Patterns and Breastfeeding: Nationwide Clustering Study
Source: Pharmacoepidemiol Drug Saf. 2026 Aug 30;35(9):e70470. doi: 10.1002/pds.70470 (PMC13526646; doi:10.1002/pds.70470)
Supplement: Supplementary file 1 [file PDS-35-e70470-s001.docx]

**Supplementary Materials**

**Postpartum psychotropic treatment patterns and breastfeeding: nationwide clustering study**

**Table S1.** Tuning parameters for the six hierarchical cluster analyses (HCA) performed. The theta parameters, $\boldsymbol{\theta}=\left( {\boldsymbol{\theta}_{\mathbf{0}},\boldsymbol{\theta}}_{\mathbf{1}}, \boldsymbol{\theta}_{\mathbf{2}}, \boldsymbol{\theta}_{\mathbf{3}}, \boldsymbol{\theta}_{\mathbf{4}},\boldsymbol{\theta}_{\mathbf{5}} \right)$ encodes the relative importance of each ATC level. This study only includes psychotropics therefore only the tuning of ${\boldsymbol{\theta}_{\mathbf{2}}, \boldsymbol{\theta}}_{\mathbf{3}}, \boldsymbol{\theta}_{\mathbf{4}},\boldsymbol{\theta}_{\mathbf{5}}$ is important. It is also possible to tune the HCA by gamma (**γ**), which controls the importance of the dose-timing aspect and the ATC of the psychotropic treatment pattern. A larger **γ** gives more weight to the timing aspect.

|  | $\boldsymbol{\theta}_{\boldsymbol{0}}$ | $\boldsymbol{\theta}_{\boldsymbol{1}}$ | $\boldsymbol{\theta}_{\boldsymbol{2}}$ | $\boldsymbol{\theta}_{\boldsymbol{3}}$ | $\boldsymbol{\theta}_{\boldsymbol{4}}$ | $\boldsymbol{\theta}_{\boldsymbol{5}}$ | $\boldsymbol{\gamma}$ |
| --- | --- | --- | --- | --- | --- | --- | --- |
| Hierarchical cluster analysis 1 (Presented in article) | 1.00 | 1.00 | 0.50 | 0.25 | 0.10 | 0.00 | 1.00 |
| Hierarchical cluster analysis 2 | 1.00 | 1.00 | 0.50 | 0.25 | 0.10 | 0.00 | 2.00 |
| Hierarchical cluster analysis 3 | 1.00 | 1.00 | 0.60 | 0.40 | 0.20 | 0.00 | 1.00 |
| Hierarchical cluster analysis 4 | 1.00 | 1.00 | 0.60 | 0.40 | 0.20 | 0.00 | 2.00 |
| Hierarchical cluster analysis 5 | 1.00 | 1.00 | 0.85 | 0.65 | 0.40 | 0.00 | 1.00 |
| Hierarchical cluster analysis 6 | 1.00 | 1.00 | 0.85 | 0.65 | 0.40 | 0.00 | 2.00 |

**Table S2.** Prescribed Daily Doses used in the study

| ATC | Drug name (WHO) | PDD (Prescribed Daily Dose) |
| --- | --- | --- |
| N06AA09 | amitriptyline | 150 mg |
| N06AB04 | citalopram | 20 mg |
| N06AB06 | sertraline | 100 mg |
| N05BA01 | diazepam | 20 mg |
| N06AX16 | venlafaxine | 225 mg |
| N05BA12 | alprazolam | 1 mg |
| N05AF03 | chlorprothixene | 50 mg |
| N05CH01 | melatonin | 2 mg |
| N05CD05 | triazolam | 0.5 mg |
| N06AX22 | agomelatine | 50 mg |
| N05AH04 | quetiapine | 25 mg |
| N03AX09 | lamotrigine | 300 mg |
| N06AA10 | nortriptyline | 150 mg |
| N05BA04 | oxazepam | 15 mg |
| N06AX03 | mianserin | 60 mg |
| N05CF01 | zopiclone | 7.5 mg |
| N06AB05 | paroxetine | 40 mg |
| N06AX11 | mirtazapine | 60 mg |
| N05CF02 | zolpidem | 10 mg |
| N06BA04 | methylphenidate | 40 mg |
| N05AN01 | lithium | 24 mmol |
| N05AB03 | perphenazine | 36 mg |
| N06AB03 | fluoxetine | 40 mg |
| N06AB10 | escitalopram | 20 mg |
| N05AH03 | olanzapine | 15 mg |
| N05CD06 | lormetazepam | 2 mg |
| N06AA02 | imipramine | 150 mg |
| N05AX08 | risperidone | 4 mg |
| N06BA09 | atomoxetine | 80 mg |
| N05CD02 | nitrazepam | 10 mg |
| N06AX12 | bupropion | 150 mg |
| N05AX13 | paliperidone | 6-12 mg (6 mg used in analyses) |
| N06AX21 | duloxetine | 120 mg |
| N05BB01 | hydroxyzine | 50-100 mg (50 mg used in analyses) |
| N06AA04 | clomipramine | 150 mg |
| N05AX12 | aripiprazole | 15 mg |
| N03AG01 | valproic acid | 1500 mg |
| N05AF05 | zuclopenthixol | 20-40 mg (20 mg used in analyses) |
| N06BA07 | modafinil | 200 mg |
| N05BA09 | clobazam | 30 mg |
| N05AF01 | flupentixol | 6 mg |
| N06AA12 | doxepin | 150 mg |
| N05BE01 | buspirone | 30 mg |
| N05AE04 | ziprasidone | 80 mg |
| N05BA02 | chlordiazepoxide | 30 mg |
| N05AA02 | levomepromazine | 300 mg |
| N05BA06 | lorazepam | 3 mg |
| N05AL05 | amisulpride | 600 mg |
| N05CF03 | zaleplon | 10 mg |
| N05AD05 | pipamperone | 120 mg |
| N05CD08 | midazolam | 15 mg |
| N06BA02 | dexamfetamine | 20 mg |
| N05BA08 | bromazepam | 12 mg |
| N03AE01 | clonazepam | 1.5 mg |
| N03AX11 | topiramate | 200 mg |
| N05AB04 | prochlorperazine | 15 mg |
| N05AD03 | melperone | 100 mg |
| N05AL01 | sulpiride | 600 mg |
| N06AX26 | vortioxetine | 20 mg |
| N06BA12 | lisdexamfetamine | 50 mg |
| N05AH02 | clozapine | 400 mg |
| N05AD01 | haloperidol | 10 mg |
| N06AB08 | fluvoxamine | 150 mg |
| N06AX18 | reboxetine | 8 mg |
| N03AF01 | carbamazepine | 800 mg |
| N05AG02 | pimozide | 4 mg |
| N05AH05 | asenapine | 10 mg |
| N05AE03 | sertindole | 16 mg |
| N06AA16 | dosulepin | 150 mg |
| N03AX14 | levetiracetam | 1500 mg |
| N05AE05 | lurasidone | 74 mg |
| N03AX18 | pregabalin | 300 mg |
| N06AF01 | isocarboxazid | 30 mg |
| N05AX15 | cariprazine | 3 mg |
| N05AX16 | brexpiprazole | 2 mg |
| N03AB02 | phenytoin | 300 mg |
